# A peptide derived from HSP60 reduces proinflammatory cytokines and soluble mediators: a therapeutic approach to inflammation

**DOI:** 10.3389/fimmu.2023.1162739

**Published:** 2023-04-28

**Authors:** Maria del Carmen Domínguez-Horta, Anabel Serrano-Díaz, Mabel Hernández-Cedeño, Gillian Martínez-Donato, Gerardo Guillén-Nieto

**Affiliations:** ^1^ Autoimmunity Project, Pharmaceutical Division, Center for Genetic Engineering and Biotechnology, Havana, Cuba; ^2^ Physiology Department, Latin American School of Medicine, Havana, Cuba; ^3^ Biomedical Research Division, Center for Genetic Engineering and Biotechnology, Havana, Cuba

**Keywords:** HSP60, APL, cytokines, inflammation, rheumatoid arthritis, CIGB-814/CIGB-258, COVID-19, Jusvinza

## Abstract

Cytokines are secretion proteins that mediate and regulate immunity and inflammation. They are crucial in the progress of acute inflammatory diseases and autoimmunity. In fact, the inhibition of proinflammatory cytokines has been widely tested in the treatment of rheumatoid arthritis (RA). Some of these inhibitors have been used in the treatment of COVID-19 patients to improve survival rates. However, controlling the extent of inflammation with cytokine inhibitors is still a challenge because these molecules are redundant and pleiotropic. Here we review a novel therapeutic approach based on the use of the HSP60–derived Altered Peptide Ligand (APL) designed for RA and repositioned for the treatment of COVID-19 patients with hyperinflammation. HSP60 is a molecular chaperone found in all cells. It is involved in a wide diversity of cellular events including protein folding and trafficking. HSP60 concentration increases during cellular stress, for example inflammation. This protein has a dual role in immunity. Some HSP60-derived soluble epitopes induce inflammation, while others are immunoregulatory. Our HSP60-derived APL decreases the concentration of cytokines and induces the increase of FOXP3+ regulatory T cells (Treg) in various experimental systems. Furthermore, it decreases several cytokines and soluble mediators that are raised in RA, as well as decreases the excessive inflammatory response induced by SARS-CoV-2. This approach can be extended to other inflammatory diseases.

## 1 Introduction

Cytokines are molecules with relatively low molecular weights. These proteins are secreted by several immune cells (1, 2). Some cytokines are pro-inflammatory and others are anti-inflammatory. Consequently, they act mostly as regulators of immune and inflammatory responses (3–5).

Additionally, cytokines have important functions in a diversity of biological events including cell activation, differentiation, and proliferation (6, 7). Furthermore, cytokines play a crucial role in acute and chronic inflammation, and tumor progression, as well as in the onset and chronicity of autoimmune diseases (1, 6, 8).

The inhibition of proinflammatory cytokines has been used in the treatment of autoimmune diseases such as RA (9–11). Some of these drugs were repositioned in the management of COVID-19 patients (12, 13). However, controlling hyperinflammation with cytokine inhibitors is still a challenge, because these molecules are redundant and pleiotropic (14, 15). Likewise, these drugs are immunosuppressive and they can lead to the fall of the general condition of COVID-19 patients (16–18).

In this article, we focus on an original approach to the control of inflammation using an APL derived from HSP60. This therapeutic concept is focused on the induction of peripheral tolerance using a modified autoantigen implicated in RA pathogenesis. HSP60 was the autoantigen chosen for the APL design. Interestingly, this protein plays a dual role in immunity, i.e. certain soluble HSP60-derived epitopes induce inflammation and others are immunoregulatory (19–23).

The APL designed has anti-inflammatory properties and increases Treg in preclinical models of RA (24, 25). Clinical investigations in RA patients indicate that this molecule is safe and reduces inflammation (26–28).

Based on these results we decided to investigate whether this molecule could be useful in treating the hyperinflammation that distinguishes COVID-19 patients who are progressing to severe and critical conditions. The treatment with this peptide inhibited several cytokines and soluble mediators associated with hyperinflammation in COVID-19 patients (29, 30).

Given all these results, the anti-inflammatory effects of this molecule are assessed in other experimental models of inflammation.

## 2 HSP60, autoantigen selected for APL design

Among the autoantigens involved in RA pathogenesis, we selected HSP60 for the APL design. This selection was supported by the therapeutic potential of HSP60 for autoimmune diseases. We hypothesized that an APL derived from HSP60 would enhance these therapeutic effects.

HSP60 is extremely conserved in evolution (31, 32). This protein is classified as a chaperone and is located inside the mitochondria where, together with the co-chaperonin Hsp10, it assists in protein homeostasis (33, 34).

However, under certain physiological conditions, HSP60 is located in other cellular organelles and it can even appear in the extracellular space. At these sites, HSP60 participates in pathogenic events such as inflammation, autoimmunity and carcinogenesis (35, 36).

HSP60 has an interesting connection with innate and acquired immune response, which is related to its conservation through evolution and its chaperone function.

This molecule has been described as an innate signal for macrophages and dendritic cells. Macrophages in reaction to HSP60 produce pro-inflammatory molecules (37–39). HSP60 stimulates the maturation of dendritic cells (40).

This chaperone was identified as a dominant bacterial antigen during infections or vaccination. Both biological events are characterized by the secretion of antibodies to bacterial HSP60 (41). Moreover, autoantibodies to self-HSP60 are identified in several autoimmune diseases such as RA, lupus, inflammatory bowel disease and atherosclerosis (42–46).

Furthermore, autoantibodies against HSP60 are present in healthy subjects; for example, an Ig M iso-type autoantibody against HSP60 was identified within the blood of the umbilical cord of some newborns (47), and IgM and IgG autoantibodies against HSP60 were identified in healthy human beings (48).

On the other hand, epitopes derived from mycobacterial HSP65 that are identical to the HSP60 human peptide, induced cytotoxic T cell reactivity in healthy humans (20). Besides, T cells against HSP60 self-peptides were found in the blood of the umbilical cord of healthy neonates (39). These facts indicate that it is normal to find effector T cells to self-HSP60 from birth.

Cohen hypothesized that HSP may be included in the “immunological homunculus”, which comprises several dominant antigens involved in an intricate biologic regulatory network (40).

Other authors have described that T cells to self-HSP60 are related to spontaneous remission in juvenile idiopathic arthritis (41, 42) and induce resistance to induction of experimental arthritis in Lewis rats (43–45). T cells against HSP60 that secreted IL-10 can be favorable in attenuating inflammation during autoimmune diseases and harmful in infections (42, 46, 47). In contrast, T cells vs HSP60 that produce proinflammatory cytokines can be damaging in autoimmune diseases and beneficial against pathogens (48).

Studies about HSP60 have progressively increased in recent years, mainly because of its potential as an approach for emerging therapeutic procedures in inflammatory diseases and severe chaperonopathies such as several kinds of cancer, as well as inflammatory and autoimmune diseases and neurodegenerative diseases (36, 49–52).

Particularly, since the regulatory effect of HSP60 on the immune response is well defined, several authors have proposed different approaches for its use in the treatment of autoimmune diseases (22, 23). Regardless of the potential risks of generating adverse inflammatory effects, diverse formulations of HSP60 and peptides derived from it, have been studied in experimental models without observing pathological autoimmunity (53).

Furthermore, reports show the use of the immunomodulatory properties of HSP60 and its peptides in clinical trials for autoimmune diseases (54–57). However, none of these therapeutic candidates have become registered drugs.

We believe that there are key points in the success of the treatments based on peptides derived from HSP60 for their use in inflammation and the reduction of pro-inflammatory cytokines, i.e., the selection of a specific epitope, the biodistribution of this molecule and the frequency of its administration.

## 3 An APL designed from HSP60 as an inductor of peripheral tolerance

The identification of an epitope from HSP60 is a critical factor for induced peripheral tolerance as a possible treatment for autoimmune diseases.

The potential of the APLs as tolerance inductors has been previously reported (58–60). APLs are similar to the wild-type peptide but with one or two mutations in the essential interaction positions with the T-cell receptor (TCR) or with the HLA class II molecules that modify the pathways for the activation of T cells. These APL can modify the response of autoreactive T cells by several mechanisms (60–64).

**Figure 1:**
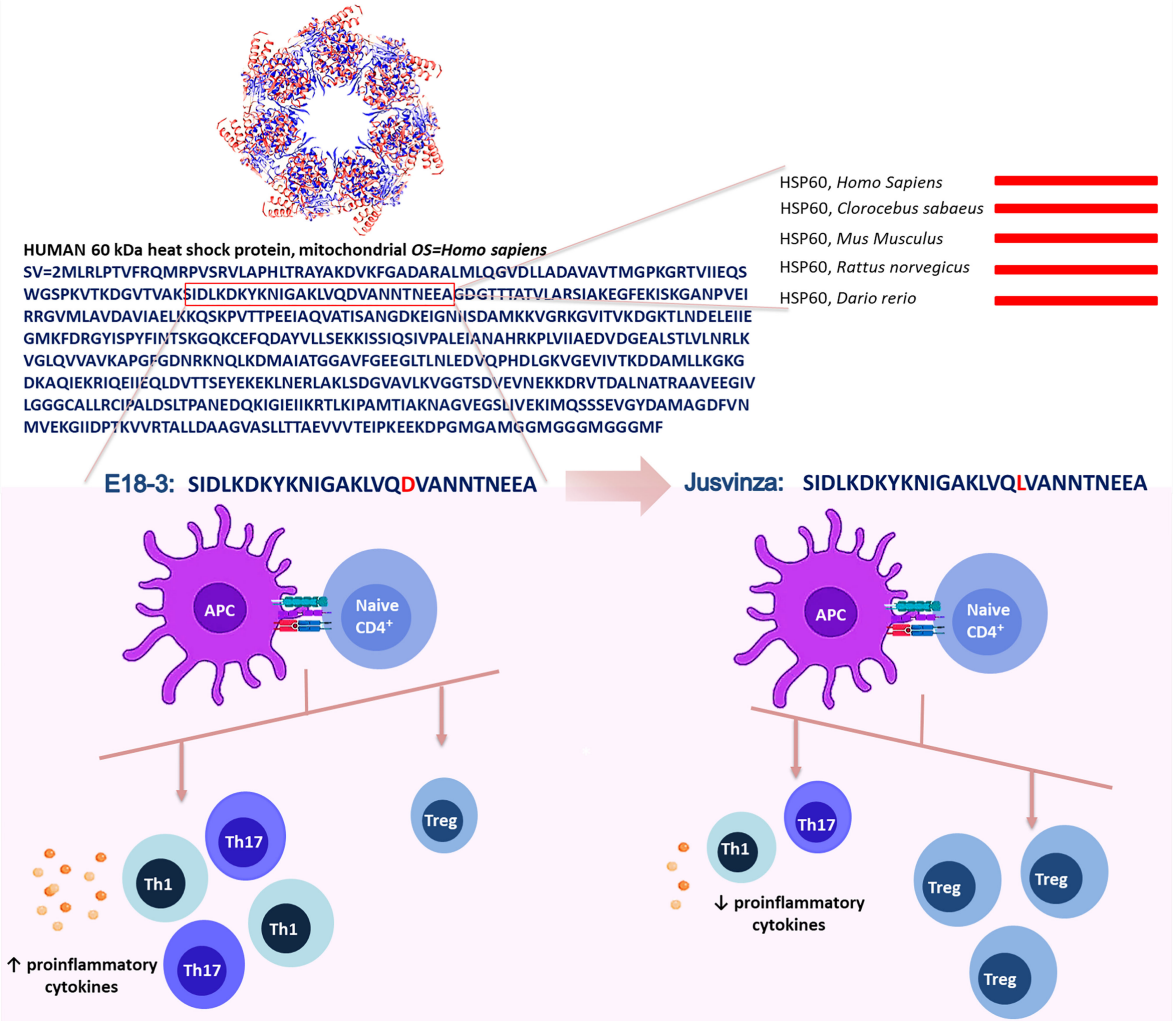
Jusvinza was designed from the N-terminal region of the human HSP60 (amino acids from 90 to 109). This sequence is 100% identical between humans, rats, mice, monkeys and Zebrafish (lines red). In this region, the aspartic acid-18 (red) involved in the interaction with the HLA class II molecule was substituted by leucine (red). This change increased the affinity between Jusvinza and HLA class II molecules. Jusvinza induces an increase of FOXP3+ regulatory T cells (Treg) and reduces proinflammatory cytokines. In contrast, the wild-type peptide did not induce Treg cells and increase proinflammatory cytokines.

In contrast with other authors, we selected the N-terminal sequence from human HSP60 (amino acids 90 to 109). This region is much conserved, it is 100% identical in humans, monkeys, fish, rats and mice, but the match is 50% with *Mycobacterium tuberculosis *(*Mt*). In this sequence, the Propred computer algorithm (65) predicted new epitopes that would be interacting directly with the HLA class II molecule associated with RA. Aspartic acid 18 was substituted by leucine (**Figure 1**). This mutation increased the affinity between APL and HLA class II molecules, according to the bioinformatic prediction (24).

Furthermore, potential binding motifs (cores) of this APL and its wild type peptide (E18-3), and the affinity of both to HLA class II alleles related with RA, were analyzed by the NetMHCIIpan platform (66, 67). This algorithm indicated that APL has two possible overlapping epitopes. A notable feature is that the replacement of Asp-18 in E18-3 with a Leu raises the affinity of APL to HLA class II. Predictions using NetMHCIIpan advise that the novel peptide binds to more RA related HLA class II molecules and has a better affinity than E18-3 (68).

This novel peptide was called APL-1 or CIGB-814 in preclinical and clinical studies in RA. This peptide was renamed CIGB-258 during clinical research in COVID-19 patients. Subsequently, CIGB-258 was granted Authorization for Emergency Use (AEU) by the Cuban Regulatory Authority for COVID-19 patients under the commercial name of Jusvinza.

## 4 Biodistribution and pharmacokinetics (PK) of Jusvinza

The biodistribution profile of a drug is essential in activating the molecular mechanisms that induced peripheral tolerance. Hence, depending on the organ where the drug is positioned, it may be able to interact with antigen-presenting cells (APC), T cells and cytokines that mediate the induction of tolerance.

**Figure 2:**
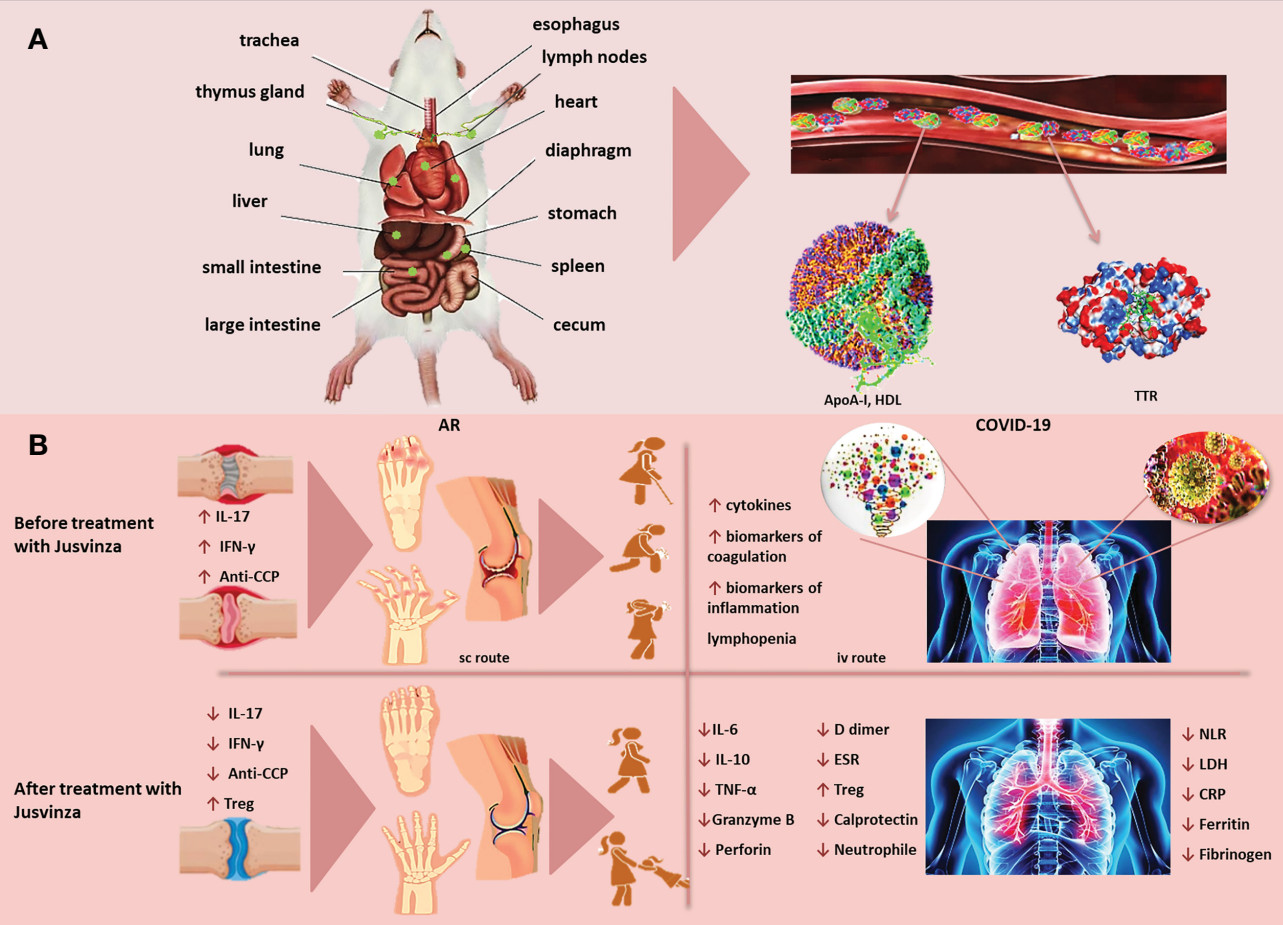
**(A)** Jusvinza is mainly distributed to the stomach and small intestine, as early as 4 hours post-administration. The concentration of Jusvinza increased in lymph nodes (LNs) at 24 hours post-administration. This peptide was also found in the liver, spleen, heart and lungs in Lewis rats. Jusvinza binds apolipoprotein A-I (Apo-AI) and transthyretin (TTR) in plasma. **(B)** Jusvinza reduces inflammation in animal models and RA patients. The processing and presentation of this APL by the antigen-presenting cells (APC) to the autoreactive T lymphocytes in the periphery could induce the expansion of Treg. These cells migrate to the swollen joints and attenuate autoreactive T cells responsible for arthritis pathogenesis. In addition, Jusvinza reduces TNFα, IL-17, interferon-gamma (IFN-γ), anti-CCP antibodies and neutrophil migration. Jusvinza reduces hyperinflammation in COVID-19 patients. Jusvinza can inhibit the activity of monocytes, macrographs and neutrophils. This inhibition may contribute to the decrease of IL-6, TNFa and IL-10, as well as the restoration of lymphocyte counts in patients. Additionally, this peptide can induce Treg. These activated cells migrate to inflamed sites and could cross-recognize the wild-type epitope from HSP60 expressed in the endothelial tissue and inhibit autoimmune damage induced during viral infection.

The biodistribution of a peptide can be affected by the dose, as well as by the inoculation route. Jusvinza was mainly distributed in the stomach and small intestine, after being inoculated in Lewis rats. The levels of Jusvinza increased in lymph nodes (LNs) at 24 hours, compared to four hours postadministration. This peptide was likewise found in the liver, spleen, heart and lungs (**Figure 2A**). The biodistribution of Jusvinza was almost the same for the three routes studied (subcutaneous, intravenous and intradermal routes) (69). This biodistribution profile is interesting because the small intestine is specialized in the induction of tolerance (70).

In addition, the highest concentration of Jusvinza in the blood plasma of rats was found at 0.5 to 1 hour; and the half-life in the blood was calculated to be of six hours. These results agree with the PK profile identified in RA patients included in the Phase I Clinical Trial (27).

Recently, we have identified that Jusvinza binds only with apolipoprotein A-I (Apo-AI) and transthyretin in human plasma. The identification of these proteins were through affinity chromatography using as the matrix a pearl-shaped resin (ChemMatrix) - coupled to Jusvinza. The eluted proteins were accurately identified by mass spectrometry (nano ESI-MS). Apo-AI and transthyretin are involved in lipid metabolism, and changes in their conformation and concentration have been associated with diseases such as type II diabetes and atherosclerosis. These results agree with those of Cho et al, who demonstrated that Jusvinza enhanced HDL stability (71).

Future research is needed to elucidate the biological significance of the binding of Jusvinza to Apo-AI and transthyretin.

## 5 Jusvinza reduces inflammation and TNFα in two animal models for RA

The biological effect of Jusvinza was assessed in an experimental model in rats (72). Jusvinza has an epitope that could be bound to rat MHC class II molecules (RT1.BI); according to the MHC2PRED software (73). In this animal model, the disease is induced in rats through immunization with *Mt* in incomplete Freund’s adjuvant (AA). Jusvinza reduced the inflammation and pannus in these rats with arthritis.

However, the original epitope from HSP60 did not produce these therapeutic effects in rats. Besides, the treatment with Jusvinza significantly reduces TNFα levels in the spleen of treated animals compared to the placebo group. This clinical efficacy induced by Jusvinza was associated with an increase of Treg in the rats.

The AA model is characterized by the fact that immunization with *Mt* protects against subsequent attempts to induce this disease. This effect is due to the induction of Treg against a conserved epitope from HSP60 (aa from 256 to 270) and the secretion of regulatory cytokines (41, 74). These factors could favor the therapeutic effect of Jusvinza in the AA model. Hence, it was essential to assess the therapeutic effect of Jusvinza in another animal model, in which Treg cells are not induced against HSP60 in the course of the disease.

Consequently, the therapeutic effect of Jusvinza was evaluated in Collagen Induced Arthritis (CIA) in DBA/1 mice (75). Jusvinza monotherapy was able to reduce inflammation in these mice. The therapeutic effect was similar to mice inoculated with Jusvinza plus methotrexate (MTX) (25). Furthermore, pannus development was not detected in mice treated with Jusvinza. This indicates that the migration of macrophages and neutrophils did not take place in the joints of mice treated with this peptide.

These results were also linked to a decrease in TNFα levels. This cytokine is crucial for the onset and chronicity of RA. TNFα can stimulate proinflammatory cytokine production, increasing the expression of adhesion molecules and neutrophil activation, and enhancing antibody secretion by plasmatic cells (76–78).

Contrary to the effect in the spleen of AA rats treated with Jusvinza, Treg was not detected in CIA mice. Nevertheless, the treatment with Jusvinza plus MTX induced Treg in the spleen of CIA mice. The molecular mechanism of MTX is not associated with an induction of Treg in mice (79). But, the decrease of TNFα mediated by MTX can favor the induction of Treg by Jusvinza. The results suggest that MTX plus Jusvinza could have a molecular synergic effect in the CIA model.

Jusvinza reduced inflammation in AA and CIA models mediated by the expansion of Treg at the periphery. These cells could migrate to the swollen joints and decrease autoreactive T cell activity that perpetuates arthritis, blocking the production of proinflammatory cytokines and the consequent neutrophil migration.

## 6 Jusvinza induces Treg with suppression activity and reduces IL-17 secreted by Th17

Jusvinza induced Treg in draining lymph nodes and spleen from healthy BALB/c mice. At the same time, the wild-type epitope of HSP60 recruits more CD4+ FoxP3- T lymphocytes, showing that the mutation of the wild-type peptide was effective in inducing Treg and reinforces the beneficial potentials of Jusvinza for the management of RA patients (24).

In addition, experiments with peripheral blood mononuclear cells (PBMC) from RA patients indicate that Jusvinza induces Treg (24, 80). In contrast, the wild-type peptide did not induce Treg (24).

Furthermore, autologous cross-over experiments showed that Jusvinza-treatment had a significant effect in reducing IL-17 levels. Jusvinza can induce Tregs and its suppressive activity against antigen-specific T lymphocytes cells, whereas the activated T effector cells produce less IL-17 (68).

The change of sequence produced in Jusvinza could increase Treg in RA patients and specifically inhibit autoreactive T cells. Treatment with Jusvinza could help restore the healthy Th17/Treg balance for patients with RA and other autoimmune diseases.

## 7 Jusvinza reduces inflammation, proinflammatory cytokines and autoantibodies against cyclic citrullinated peptides (anti-CCP) in RA patients

The safety of Jusvinza was assessed in patients with RA, during an open phase I clinical trial. Twenty patients with moderately active RA were involved in this study. Three doses (1, 2.5 and 5 mg) of Jusvinza were evaluated by the subcutaneous route. This study included the restriction of the use of non-steroidal anti-inflammatory drugs, disease-modifying anti-rheumatic drugs and corticosteroids, as of four weeks before the start of the Jusvinza treatment. Clinical response in patients was assessed following the guidelines of the American College of Rheumatology and Disease Activity Score in 28 joints. Function and health-related quality of life, quantification of inflammatory biomarkers and radiographic changes in patients were also evaluated.

Jusvinza was well tolerated at all doses. The adverse effects detected were minor and reversible, essentially irritation at the inoculation site. Treatment with this peptide diminished disease activity and magnetic resonance imaging score in patients. This treatment enhanced the health-related quality of life of all patients included in the study. Moreover, Jusvinza significantly reduced IL-17 and interferon-gamma (IFN-γ) in patients (26).

RA is mediated by autoreactive T lymphocytes, with TH1 and TH17 phenotypes. However, antibodies against citrullinated self-proteins have a pathogenic role in this disease. Anti-CCP antibodies are related to a fast course of RA, primary erosive damage, increased inflammation and disability in patients (80).

Jusvinza induced a significant reduction of anti-CCP antibodies in patients (28). These results suggest that Jusvinza could inhibit B lymphocytes that produce these pathogenic antibodies.

Another explanation is related to the possible induction of Treg by Jusvinza in patients. This peptide increases Tregs in some experimental models (24, 25). Tregs have different mechanisms of action that may depend on cell contact, through which they can induce apoptosis on effector T cells and plasma cells secreting anti-CCP antibodies (81). Interestingly, Jusvinza decreased the viability of PBMC isolated from RA patients by inducing apoptosis (82).

Furthermore, Jusvinza might affect the citrullination process by decreasing IL-17. Interactions with viruses or bacteria that activate NETosis are the leading sources of protein citrullination in RA (83). NETosis is a type of regulated cell death dependent on the formation of neutrophil extracellular traps. Some studies describe that HSP60 disturbs the effector functions of neutrophils, i.e. HSP60 enhances the phagocytic activity of neutrophils (84); bacterial HSP GroEL from *Staphylococcus epidermidis* biofilms promoted ADN decondensation and induced NETosis (85); inflammatory cytokines can lead to NETosis in neutrophils from RA patients (86). Specifically, IL-17 has widespread inflammatory effects on the joints, inducing bone and cartilage erosions and promoting the migration of inflammatory molecules to the synovia (87). This cytokine plays an important role in the regulation of anti-citrullinated protein antibody secretion (88).

Jusvinza decreases IL-17 and soluble mediators as anti-CCP antibodies, and it can stimulate the activation of the suppressive activity of Tregs (24, 68) (**Figure 2B**). Therefore, all results confirm the beneficial effect of Jusvinza and its possible medical application in the reduction of inflammation in RA and other inflammatory diseases.

## 8 Jusvinza reduces cytokines involved in the “cytokine storm” and soluble mediators of inflammation in COVID-19 patients

The clinical spectrum of COVID-19 is very widespread and complex. Patients may range from asymptomatic cases to those showing a rapid progression toward acute respiratory distress syndrome (ARDS) and death (89). Patients progressing to severe stages present an exacerbated inflammatory response, evidenced by the increase in the serum concentration of inflammation biomarkers (90). A group of these patients progresses to cardiovascular collapse, multiple organ failure, and death. Under these conditions, the treatments used for inflammatory chronic diseases have been repositioned to reduce hyperinflammation in COVID-19 patients (91, 92).

The anti-inflammatory effects of Jusvinza in several experimental models of RA are associated with TNFα reduction and Treg induction. At the same time, the therapeutic effect and the reduction of IL-17, IFNγ and autoantibodies against citrullinated self-proteins in RA patients (24, 26, 28, 68, 80) were the rational bases in the proposal of this molecule for the management of hyperinflammation in COVID-19 patients. Consequently, the Cuban Regulatory Authority approved the exploratory use of this peptide for COVID-19 patients in critical and serious conditions.

Exploratory studies in COVID-19 patients revealed that this APL promotes clinical and radiological improvement linked to a decrease in systemic inflammation biomarkers and IL-6, TNFα and IL-10 (29, 93). The treatment with this peptide was granted the AEU by the Cuban Regulatory Authority for COVID-19 patients (94). After this AEU, Jusvinza was included in the Cuban guidelines for the management of COVID-19 patients with signs of hyperinflammation (95).

High levels of IL-6, associated with disease severity, have been widely described in COVID-19 patients (96, 97). IL-6 is considered one of the most important cytokines in infections, along with IL-1 and TNF-α (98). Jusvinza reduced IL-6 levels linked to the clinical improvement of patients (29).

TNF-α stimulates an ongoing inflammatory response associated with autoimmune diseases (99, 100). TNF-α is a pro-inflammatory cytokine that can support T cell apoptosis by interacting with its receptor (101, 102). This cytokine is essential in the pathogenesis of lung fibrosis and SARS-CoV-2 infection (103). Interestingly, TNF-α is significantly reduced with Jusvinza therapy (29).

On the other hand, critically ill COVID-19 patients display an increase of IL-10 related to a worsening of this disease (104). IL-10 is a cytokine with many pleiotropic properties in immunoregulation and inflammation (105). The coronaviruses have intricate mechanisms that use the immunoregulatory function of IL-10 for immune evasion, helping virus replication (106). An association between a high concentration of IL-10 with the fall and functional exhaustion of CD8+ and CD4+T cells has been found in COVID-19 patients (107), indicating that this cytokine plays a key role in SARS-CoV-2 pathogenesis. The Jusvinza treatment led to IL-10 reduction after 96 hours of therapy. This reduction was associated with the clinical improvement of patients (29). IL-10 is secreted by macrophages, monocytes and T cells. However, T cells can not contribute to the high concentration of IL-10 because these patients are characterized by a marked lymphopenia, typical of COVID-19. Jusvinza diminishes monocyte and macrophage counts under cell stress (82). This is possibly the mechanism through which IL-10 is reduced in COVID-19 patients treated with Jusvinza. Besides, the reduction of IL-6 and TNF-α could downregulate IL-10.

Furthermore, high concentrations of calprotectin (S100A8/A9) are associated with negative clinical results in COVID-19 patients (108). Jusvinza reduces calprotectin in sera from COVID-19 patients and this reduction was correlated with the contraction in neutrophil count. These results are interesting because calprotectin is also increased in patients with chronic inflammatory diseases, inducing cytokine production (109).

Lymphopenia, with significantly reduced numbers of T lymphocytes, has been described in severe COVID-19 patients. This lymphopenia is related to the functional activities of cytotoxic T lymphocytes in COVID-19. Natural killer cells, total T cells, as well as CD8+ T cells, are lower in patients showing severe disease than in healthy persons (110).

Likewise, Granzyme B and perforin are increased in CD8+ T cells in severely ill patients (111). Both proteins decreased in the serum 96 hours after the treatment with Jusvinza. This reduction was related to the stabilization of lymphocyte and neutrophil counts in the patients (29).

Additionally, Tregs are significantly reduced in severely ill COVID-19 patients (112, 113). Jusvinza induces Tregs in seriously ill COVID-19 patients (29). Gammazza et al. identified an epitope from HSP60 that is common with the SARS-CoV-2 replicase polyprotein 1ab. These authors suggested that post-translational modifications during metabolic stress produced by hypertension and diabetes could disturb HSP60 localization and induce an endothelial injury (114). The beneficial result of Jusvinza in COVID-19 patients could be facilitated by the induction of Treg. These cells migrate to inflamed tissues and could cross-recognize the wild-type peptide from HSP60. This new “cross-talk” could induce regulatory mechanisms that would reduce autoimmune damage in the endothelium produced by viral infection. Moreover, Jusvinza could inhibit the activity of neutrophils, monocytes and macrographs. This inhibition may influence the decrease of several proinflammatory cytokines and soluble mediators of inflammation, as well as the increase of lymphocyte counts (**Figure 2B**). All these effects could resolve exacerbated inflammation and contribute to a positive outcome for the patients.

These results are the premise for the evaluation of the anti-inflammatory effect of Jusvinza in other experimental models of inflammation.

## 9 Anti-inflammatory activity of Jusvinza against acute toxicity induced by carboxy methyl lysine in Zebrafish

High levels of inflammatory cytokines have characterized severe stages of COVID-19 (113), autoimmune diseases and inflammatory chronic diseases (115). The non-enzymatic glycation of proteins and carbohydrates induces some glycation end products, related to hyperinflammation (116). The glycation of high-density lipoproteins (HDL) is associated with the dysfunctional activity of this lipoprotein (117), which could enhance inflammation. Glycated HDL were found to be toxic in different human cells (118, 119), and in Zebrafish and its embryos, producing reactive oxygen species and showing a slower development rate (120).

Zebrafish (*Danio rerio*) is a routinely used model to assess the toxicity of advanced glycation end products (120) and to evaluate the anti-inflammatory effect of therapeutic candidates.

Recently, Cho et al. studied the therapeutic effect of Jusvinza against inflammation induced by N-”-carboxymethyl lysine (CML) in Zebrafish embryos and adults (71). High concentrations of CML have been identified in the serum of patients with diabetes mellitus and atherosclerosis (121, 122). These patients also showed extremely high inflammatory cytokines, such as IL-1 and TNFα, indicating that a high CML concentration is linked to a pro-inflammatory condition (123). CML produced an important glycation and aggregation of HDL that disturbs the structure and function of HDL, which is linked to a reduction in apolipoprotein A-I stability (118).

Microinjection of CML in Zebrafish embryos produces high embryonic death rates, where survival is of only 18% of the fish showing developmental defects. However, Jusvinza co-injection induces a significant increase in survival and normal development of the fish. Furthermore, an intraperitoneal inoculation of CML in adult Zebrafish produced acute paralysis, sudden death, and affected its swimming capacity through hyperinflammation. But, a co-inoculation of Jusvinza caused a quicker recovery of swimming capacity and a higher survivable rate. Interestingly, the group treated only with CML showed a survival rate of 49%, while the group treated with CML and inoculated with Jusvinza had a survival rate of 97%, with a significant decrease in liver inflammation.

On the other hand, these authors compared the efficacy of Jusvinza, Infliximab (Remsima®), and Tocilizumab (Actemra®) in the model of the acute death of Zebrafish induced by CML. The results showed that the Jusvinza group had a quicker recovery and swimming capacity, with a higher survivable rate than the Remsima® group (71).

## 10 Conclusions and perspectives

This review describes an important translational research outcome; from the design of an APL for the induction of tolerance in RA, followed by its assessment in experimental models, and finally the treatment of the inflammation that characterizes RA and COVID-19 patients.

The anti-inflammatory effect of Jusvinza constitutes an attractive therapeutic approach for an extensive variety of diseases, characterized by inflammation and high levels of proinflammatory cytokines, such as autoimmune diseases, COVID-19, atherosclerosis, diabetes and neurodegenerative diseases.

## Author contributions

Conceptualization: MdCD-H; Writing – original draft: MdCD-H, AS-D, and MH-C; Writing – review & editing: MdCD-H, AS-D, MH-C, GM-D, and GG-N. All authors contributed to the article and approved the submitted version.

## References

[1] Stow JL Murray RZ. Intracellular trafficking and secretion of inflammatory cytokines. Cytokine Growth Factor Rev (2013) 24:227–39. doi: 10.1016/j.cytogfr.2013.04.001 23647915

[2] Gulati K Guhathakurta S Joshi J Rai N Ray A. Cytokines and their role in health and disease: a brief overview. MOJ Immunol (2016) 4:1–9. doi: 10.15406/moji.2016.04.00121

[3] Rodrigues G Bottaro G Gobbe M Maschio-Signorini LB Pires de Campos DA. Proinflammatory and anti-inflammatory cytokines mediated by NF-κB factor as prognostic markers in mammary tumors. Mediators Inflamm (2016) 2016:10. doi: 10.1155/2016/9512743 PMC4771900 26989335

[4] Azizi G Mirshafiey A. The potential role of proinflammatory and antiinflammatory cytokines in Alzheimer disease pathogenesis. Immunopharmacol Immunotoxicol (2012) 34:881–95. doi: 10.3109/08923973.2012.705292 22970774

[5] Souza K Gurgul-Convey E Elsner M Lenzen S. Interaction between pro-inflammatory and anti-inflammatory cytokines in insulin-producing cells. J Endocrinol (2008) 197:139–50. doi: 10.1677/JOE-07-0638 18372240

[6] Dinarello CA. Historical insights into cytokines. Eur J Immunol (2007) 37(Suppl. 1):S34–45. doi: 10.1002/eji.200737772 PMC3140102 17972343

[7] Lacy P Stow JL. Cytokine release from innate immune cells: association with diverse membrane trafficking pathways. Blood (2011) 118:9–18. doi: 10.1182/blood-2010-08-265892 21562044

[8] O’Shea JJ Ma A Lipsky P. Cytokines and autoimmunity. Nat Rev Immunol (2002) 2:37–45. doi: 10.1038/nri702 11905836

[9] Li P Zheng Y Chen X. Drugs for autoimmune inflammatory diseases: from small molecule compounds to anti-TNF biologics. Front Pharmacol (2017) 8:460. doi: 10.3389/fphar.2017.00460 28785220 PMC5506195

[10] Lai Y Dong C. Therapeutic antibodies that target inflammatory cytokines in autoimmune diseases. Int Immunol (2016) 28(4):181–8. doi: 10.1093/intimm/dxv063 PMC4889878 26545932

[11] Findeisen KE Sewell J Ostor AJK. Biological therapies for rheumatoid arthritis: an overview for the clinician. Biologics (2021) 15:343–52. doi: 10.2147/BTT.S252575 PMC8370108 34413630

[12] Sanghai N Shafiq K Tranmer GK. Drug discovery by drug repurposing: combating COVID-19 in the 21st century. Mini Rev Med Chem (2021) 21:3–9. doi: 10.2174/1389557520999200824103803 32838716

[13] Yousefi H Mashouri L Okpechi SC Alahari N Alahari SK Yousefi H. Repurposing existing drugs for the treatment of COVID-19/SARS-CoV-2 infection: a review describing drug mechanisms of action. Biochem Pharmacol (2021) 183:114296. doi: 10.1016/j.bcp.2020.114296 33191206 PMC7581400

[14] Moraga I Spangler J Mendoza JL Garcia KC. Multifarious determinants of cytokine receptor signaling specificity. Adv Immunol (2014) 121:1–39. doi: 10.1016/B978-0-12-800100-4.00001-5 24388212 PMC4449261

[15] Ozaki K Leonard WJ. Cytokine and cytokine receptor. pleiotropy and redundancy. J Biol Chem (2002) 277(33):29355–8. doi: 10.1074/jbc.R200003200 12072446

[16] Capraa R De Rossia N Mattiolib F Romanelli G Scarpazza C Sormani MP. Impact of low dose tocilizumab on mortality rate in patients with COVID-19 related pneumonia. Eur J Internal Med (2020) 76:31–5. doi: 10.1016/j.ejim.2020.05.009 PMC7219361 32405160

[17] Peterson D Damsky W King B. The use of janus kinase inhibitors in the time of SARS-CoV-2. J Am Acad Dermatol (2020) 82(6):e223–6. doi: 10.1016/j.jaad.2020.03.099 PMC7144601 32278797

[18] Gatti M Turrini E Raschi E Sestili P Fimognari C. Janus kinase inhibitors and coronavirus disease (COVID)-19: rationale clinical evidence and safety issues. Pharmaceuticals (2021) 14(8):738. doi: 10.3390/ph14080738 34451835 PMC8401109

[19] Cohen-Sfady M Nussbaum G Pevsner-Fischer M Mor F Carmi P Zanin-Zhorov A. Heat shock protein 60 activates b cells via the TLR4-MyD88 pathway. J Immunol (2005) 175(6):3594–602. doi: 10.4049/jimmunol.175.6.3594 16148103

[20] Munk ME Schoel B Modrow S Karr RW Young RA Kaufmann SH. T Lymphocytes from healthy individuals with specificity to self-epitopes shared by the mycobacterial and human 65-kilodalton heat shock protein. J Immunol (1989) 143(9):2844–9. 2509558

[21] Prakken BJ Roord S Ronaghy A Wauben M Albani S van Eden W. Heat shock protein 60 and adjuvant arthritis: a model for T cell regulation in human arthritis. Springer Semin Immunopathol (2003) 25:47–63. doi: 10.1007/s00281-003-0128-7 12904891

[22] Cohen IR Quintana FJ Mimran A. T Regs in T cell vaccination: exploring the regulation of regulation. J Clin Invest (2004) 114:1227–32. doi: 10.1172/JCI23396 PMC524317 15520852

[23] van Eden W van der Zee R Prakken B. Heat-shock proteins induce T-cell regulation of chronic inflammation. Nat Rev Immunol (2005) 5:318–30. doi: 10.1038/nri1593 15803151

[24] Domínguez MC Lorenzo N Barberá A Darrasse-Jeze G López N Hernández MV. An altered peptide ligand corresponding to a novel epitope from heat-shock protein 60 induces regulatory T cells and suppresses pathogenic response in an animal model of adjuvant induced athritis. Autoimmunity (2011) 44(6):471–82. doi: 10.3109/08916934.2010.550590 21370936

[25] Lorenzo N Altruda F Silengo L Dominguez MC. APL-1, an altered peptide ligand derived from heat-shock protein, alone or combined with methotrexate attenuates murine collagen induced arthritis. Clin Exp Med (2017) 17:209–16. doi: 10.1007/s10238-016-0412-7 27160252

[26] Prada D Gómez J Lorenzo N Corrales O López A González E. Phase I clinical trial with a novel altered peptide ligand derived from human heat-shock protein 60 for treatment of rheumatoid arthritis: safety, pharmacokinetics and preliminary therapeutic effects. J Clin Trials (2018) 8:1. doi: 10.4172/2167-0870.1000339

[27] Cabrales-Rico A Ramos Y Besada V Domínguez MC Lorenzo N García O. Development and validation of a bioanalytical method based on LC–MS/MS analysis for the quantitation of CIGB-814 peptide in plasma from rheumatoid arthritis patients. J Pharm BioMed Anal (2017) 143:130–40. doi: 10.1016/j.jpba.2017.05.030 28595106

[28] Corrales O Hernández L Prada D Gómez J Reyes Y Lopez AM. CIGB-814, an altered peptide ligand derived from human heat-shock protein 60, decreases anti-cyclic citrullinated peptides antibodies in patients with rheumatoid arthritis. Clin Rheumatol (2019) 38:955–60. doi: 10.1007/s10067-018-4360-3 30415439

[29] Hernández-Cedeño M Venegas-Rodríguez R Peña-Ruiz R Bequet-Romero M Santana-Sanchez R Penton-Arias E. CIGB-258, a peptide derived from human heat-shock protein 60, decreases hyperinflammation in COVID-19 patients. Cell Stress Chaperones (2021) 26(3):515–25. doi: 10.1007/s12192-021-01197-2 PMC7904296 33629254

[30] Baldomero JE Del Río A del Rosario L Venegas R Hernández M Serrano A. Early treatment with a peptide derived from the human heat-shock 60 protein avoids progression to severe stages of COVID-19. J Biotechnol BioMed (2021) 4(4):196–210. doi: 10.26502/jbb.2642-91280045

[31] Gupta RS. Evolution of the chaperonin families (Hsp60, Hsp10 and tcp- 1) of proteins and the origin of eukaryotic cells. Mol Microbiol (1995) 15:1–11. doi: 10.1111/j.1365-2958.1995.tb02216.x 7752884

[32] Marino Gammazza A Bucchieri F Grimaldi LME Benigno A Conway de Macario E Macario AJL. The molecular anatomy of human Hsp60 and its similarity with that of bacterial orthologs and acetylcholine receptor reveal a potential pathogenetic role of anti-chaperonin immunity in myasthenia gravis. Cell Mol Neurobiol (2012) 32:943–47. doi: 10.1007/s10571-011-9789-8 PMC11498446 22258649

[33] Richardson A Landry SJ Georgopoulos C. The ins and outs of a molecular chaperone machine. Trends Biochem Sci (1998) 23:138–43. doi: 10.1016/s0968-0004(98)01193-1 9584617

[34] Vilasi S Bulone D Caruso-Bavisotto C Campanella C Marino-Gammazza A San Biagio PL. Chaperonin of group I: oligomeric spectrum and biochemical and biological implications. Front Mol Biosci (2018) 4:99. doi: 10.3389/fmolb.2017.00099 29423396 PMC5788889

[35] Macario AJL Conway de Macario E. Molecular mechanisms in chaperonopathies: clues to understanding the histopathological abnormalities and developing novel therapies. J Pathol (2020) 250:9–18. doi: 10.1002/path.5349 31579936

[36] Hoter A Rizk S Naim HY. The multiple roles and therapeutic potential of molecular chaperones in prostate cancer. Cancers (2019) 11(8):1194. doi: 10.3390/cancers11081194 31426412 PMC6721600

[37] Osterloh A Kalinke U Weiss S Fleischer B Breloer M. Synergistic and differential modulation of immuneresponses by Hsp60 and lipopolysaccharide. J Biol Chem (2007) 282:4669–80. doi: 10.1074/jbc.M608666200 17164250

[38] Vabulas RM Ahmad-Nejad P da Costa C Miethke T Kirschning CJ Häcker H. Endocytosed HSP60s use toll-like receptor2 (TLR2) and TLR4 to activate the toll/interleukin-1 receptor signaling pathway in innate immune cells. J Biol Chem (2001) 276:31332–39. doi: 10.1074/jbc.M103217200 11402040

[39] Lehnardt S Schott E Trimbuch T Laubisch D Krueger C Wulczyn G. A vicious cycle involving release of heat shock protein 60 from injured cells and activation of toll-like receptor 4 mediates neurodegeneration in the CNS. J Neurosci (2008) 8(10):2320–31. doi: 10.1523/JNEUROSCI.4760-07.2008 PMC6671170 18322079

[40] Cohen IR Young DB. Autoimmunity, microbial immunity and the immunological homunculus. Immunol Today (1991) 12:105–10. doi: 10.1016/0167-5699(91)90093-9 2059311

[41] Kaufmann SH. Immunity to bacteria. Curr Opin Immunol (1989) 2:353–59. doi: 10.1016/0952-7915(89)90141-6 2534501

[42] De Kleer IM Kamphuis SM Rijkers GT Scholtens L Gordon G De Jager W. The spontaneous remission of juvenile idiopathic arthritisis characterized by CD30+Tcells directed to human heat-shock protein 60 capable of producing the regulatory cytokine interleukin-10. Arthritis Rheumatol (2003) 48:2001–10. doi: 10.1002/art.11174 12847694

[43] Anderton SM van der Zee R Prakken B Noordzij A van Eden W. Activation of T cells recognizing self 60-kD heat shock protein can protect against experimental arthritis. J Exp Med (1995) 181:943–52. doi: 10.1084/jem.181.3.943 PMC2191900 7869052

[44] Quintana FJ Hagedorn PH Elizur G Merbl Y Domany E Cohen IR. Functional immunomics: microarray analysis of IgG autoantibody repertoires predicts the future response of mice to induced diabetes. Proc Natl Acad Sci U.S.A (2004) 101(Suppl.2):14615–21. doi: 10.1073/pnas.0404848101 PMC521990 15308778

[45] Paul GA van Kooten PJ van Eden W van der Zee R. Highly autoproliferative T cells specific for 60-kDa heat shock protein produce IL-4/IL-10 and IFN γ and are protective in adjuvant arthritis. J Immunol (2000) 165:7270–7. doi: 10.4049/jimmunol.165.12.7270 11120861

[46] Xu Q Willeit J Marosi M Kleindienst R Oberhollenzer F Kiechl S. Association of serum antibodies to heat-shock protein 65 with carotid atherosclerosis. Lancet (1993) 341(8840):255–9. doi: 10.1016/0140-6736(93)92613-x 8093914

[47] Merbl Y Zucker-Toledano M Quintana FJ Cohen IR. Newborn humans manifest autoantibodies to defined self molecules detected by antigen microarray informatics. J Clin Invest (2007) 117(3):712–8. doi: 10.1172/JCI29943 PMC1804342 17332892

[48] Lang A Benke D Eitner F Engel D Ehrlich S Breloer M. Heat shock protein 60 is released inimmune-mediated glomerulonephritis and aggravates disease: *in vivo* evidence for an immunologic danger signal. J Am Soc Nephrol (2005) 16(2):383–91. doi: 10.1681/ASN.2004040276 15601747

[49] Marino Gammazza A Rizzo M Citarrella R Rappa F Campanella C Bucchieri F. Elevated blood Hsp60, its structural similarities and cross-reactivity with thyroid molecules, and its presence on the plasma membrane of oncocytes point to the chaperonin as an immunopathogenic factor in hashimoto’s thyroiditis. Cell Stress Chaperones (2014) 19:343–53. doi: 10.1007/s12192-013-0460-9 PMC3982029 24057177

[50] Meng Q Li BX Xiao X. Toward developing chemical modulators of hsp60 as potential therapeutics. Front Mol Biosci (2018) 5:35. doi: 10.3389/fmolb.2018.00035 29732373 PMC5920047

[51] Basset CA Rappa F Lentini VL Barone R Pitruzzella A Unti E. Hsp27 and Hsp60 in human submandibular salivary gland: quantitative patterns in healthy and cancerous tissues with potential implications for differential diagnosis and carcinogenesis. Acta Histochem (2021) 123:151771. doi: 10.1016/j.acthis.2021.151771 34419757

[52] Santha M Durham HD Vigh L Prodromou C. Editorial: the role of heat shock proteins in neuroprotection. Front Pharmacol (2020) 11:1227. doi: 10.3389/fphar.2020.01227 32848805 PMC7424066

[53] Prakken BJ van der Zee R Anderton SM van Kooten PJ Kuis W Van Eden W. Peptide-induced nasal tolerance for a mycobacterial heat shock protein 60 T cell epitope in rats suppresses both adjuvant arthritis and nonmicrobially induced experimental arthritis. Proc Natl Acad Sci USA (1997) 94:3284–9. doi: 10.1073/pnas.94.7.3284 PMC20361 9096385

[54] Huurman VA Decochez K Mathieu C Cohen IR Roep BO. Therapy with the hsp60 peptide DiaPep277™ in c-peptide positive type 1 diabetes patients. Diabetes Metab Res Rev (2007) 23:269–75. doi: 10.1002/dmrr.691 17024692

[55] Raz I Avron A Tamir M Metzger M Symer L Eldor R. Treatment of new-onset type 1 diabetes with peptide DiaPep277 is safe and associated with preserved beta-cell function: extension of a randomized, double-blind, phase II trial. Diabetes Metab Res Rev (2007) 23:292–8. doi: 10.1002/dmrr.712 17124720

[56] Koffeman EC Genovese M Amox D Keogh E Santana E Matteson EL. Epitope-specific immunotherapy of rheumatoid arthritis: clinical responsiveness occurs with immune deviation and relies on the expression of a cluster of molecules associated with T cell tolerance in a double-blind, placebo-controlled, pilot phase II trial. Arthritis Rheumatol (2009) 60:3207–16. doi: 10.1002/art.24916 19877047

[57] Kirkham B Chaabo K Hall C Garrood T Mant T Allen E. Safety and patient response as indicated by biomarker changes to binding immunoglobulin protein in the phase I/IIA RAGULA clinical trial in rheumatoid arthritis. Rheumatology (2016) 55:1993–2000. doi: 10.1093/rheumatology/kew287 27498355 PMC5854092

[58] Alleva DG Gaur A Jin L Wegmann D Gottlieb PA Pahuja A. Immunological characterization and therapeutic activity of an altered-peptide ligand, NBI-6024, based on the immunodominant type 1 diabetes autoantigen insulin b-chain (9–23) peptide. Diabetes (2002) 51:2126–34. doi: 10.2337/diabetes.51.7.2126 12086942

[59] Aruna BV Sela M Mozes E. Suppression of myasthenogenic responses of a T cell line by a dual altered peptide ligand by induction of CD4+CD25+ regulatory cells. PNAS (2005) 102:10285–90. doi: 10.1073/pnas.0504578102 PMC1177416 16014414

[60] Zhao J Li R He J Shi J Long L Li Z. Mucosal administration of an altered CII263-272 peptide inhibits collagen-induced arthritis by suppression of Th1/Th17 cells and expansion of regulatory T cells. Rheumatol Int (2008) 29:9–16. doi: 10.1007/s00296-008-0634-4 18600328

[61] Katsara M Deraos G Tselios T Matsoukas J Apostolopoulos V. Design of novel cyclic altered peptide ligands of myelin basic protein MBP83-99 that modulate immune responses in SJL/J mice. J Med Chem (2008) 51:3971–8. doi: 10.1021/jm8000554 18563891

[62] De Magistris M Alexander J Coggeshall M Altman A Gaeta FC Grey HM. Antigen analog-major complex histocompatibility complexes act as antagonist of the T cell receptor. Cell (1992) 68(4):625 –34. doi: 10.1016/0092-8674(92)90139-4 1739971

[63] Evavold BD Allen PM. Separation of IL-4 production from Th cell proliferation by an altered T cell ligand. Science (1991) 252:1308–10. doi: 10.1126/science.1833816 1833816

[64] Paas-Rozner M Sela M Mozes E. A dual altered peptide ligand down-regulates myasthenogenic T cell responses by up-regulating CD25- and CTLA-4-expressing CD4+ T cells. Proc Natl Acad Sci U.S.A. (2003) 100:6676–81. doi: 10.1073/pnas.1131898100 PMC164506 12743364

[65] Singh H Raghava GPS. ProPred: prediction of HLA-DR binding sites. Bioinformatics (2001) . 17:1236–7. doi: 10.1093/bioinformatics/17.12.1236 11751237

[66] Dimitrov I Garnev P Flower DR Doytchinova I. MHC class II binding prediction-a little help from a friend. J BioMed Biotechnol (2010) 2010:705821. doi: 10.1155/2010/705821 20508817 PMC2875769

[67] Lin HH Zhang GL Tongchusak S Reinherz EL Brusic V. Evaluation of MHC-II peptide binding prediction servers: applications for vaccine research. BMC Bioinf (2008) . 1(12):S22–32. doi: 10.1186/1471-2105-9-S12-S22 PMC2638162 19091022

[68] Barberá A Lorenzo N van Kooten P van Roon J de Jager W Prada D. APL-1, an altered peptide ligand derived from human heat-shock protein 60, induces selective activation of nTreg which suppress CD4+ effector T cells from rheumatoid arthritis patients. Cell Stress Chaperones (2016) 21:735–44. doi: 10.1016/j.intimp.2013.10.010 PMC4908004 27241313

[69] Domínguez MC Cabrales A Lorenzo N Padrón G González LJ. Biodistribution and pharmacokinetic profiles of an altered peptide ligand derived from heat-shock proteins 60 in Lewis rats. Cell Stress Chaperones (2020) . 25(1):133–40. doi: 10.1007/s12192-019-01054-3 PMC6985321 31802366

[70] Steimle A Frick J. Molecular mechanisms of induction of tolerant and tolerogenic intestinal dendritic cells in mice. J Immunol Res (2016) 2016:12. doi: 10.1155/2016/1958650 PMC4766351 26981546

[71] Cho KH Kim JE Nam HS Kang DJ Na HJ. Anti-inflammatory activity of CIGB-258 against acute toxicity of carboxymethyllysine in paralyzed zebrafish via enhancement of high-density lipoproteins stability and functionality. Int J Mol Sci (2022) 23(17):10130. doi: 10.3390/ijms231710130 36077532 PMC9456132

[72] Ulmansky R Cohen CJ Szafer F Moallem E Fridlender ZG Kashi Y. Resistance to adjuvant arthritis is due to protective antibodies against heat shock protein surface epitopes and the induction of IL-10 secretion. J Immunol (2002) 168:6463–9. doi: 10.4049/jimmunol.168.12.6463 12055266

[73] Lata S Bhasin M Raghava GP. Application of machine learning techniques in predicting MHC binders. Methods Mol Biol (2007) 409:201–15. doi: 10.1007/978-1-60327-118-9_14 18450002

[74] Van Eden W van der Zee R Paul AG Prakken BJ Wendling U Anderton SM. Do heat shock proteins control the balance of T cell regulation in inflammatory diseases? Immunol Today (1998) 19:303–7. doi: 10.1016/s0167-5699(98)01283-3 9666602

[75] Reche PA Glutting JP Zhang H Reinherz EL. Enhancement to the RANKPEP resource for the prediction of peptide biding to MHC molecules using profiles. Immunogenetics (2004) 56(6):405–19. doi: 10.1007/s00251-004-0709-7 15349703

[76] Scott DL Kingsley GH. Tumor necrosis factors inhibitors for rheumatoid arthritis. N Engl J Med (2006) . 355:704–12. doi: 10.1056/NEJMct055183 16914706

[77] Choy EH Panayi GS. Cytokine pathways and joint inflammation in rheumatoid arthritis. N Engl J Med (2001) 344:904–16. doi: 10.1056/NEJM200103223441207 11259725

[78] Carpentier I Coormaert B Beyaert R. Function and regulation of tumor necrosis factor type 2. Curr Med Chem (2004) 11:2205–12. doi: 10.2174/0929867043364694 15279559

[79] Montesinos MC Takedachi M Thompson LF Wilder TF Fernández P Cronstein BN. The anti-inflammatory mechanism of methotrexate depends on extracellular conversion of adenine nucleotides to adenosine by ecto-5’-nucleotidase: findings in a study of ecto-5’-nucleotidase gene-deficient mice. Arthritis Rheumatol (2007) 56:1440–5. doi: 10.1002/art.22643 17469101

[80] Domínguez MC Lorenzo N Barberá A Padrón G Torres AM Hernández MV. Therapeutic effect of two altered peptide ligands derived from the human heat shock protein 60 in experimental models of rheumatoid arthritis. Biotecnología Aplicada (2013) 30:153–6.

[81] Wang P Zheng SG. Regulatory T cells and b cells: implication on autoimmune diseases. Int J Clin Exp Pathol (2013) 6:2668–74. PMC3843247 24294353

[82] Barberá A Lorenzo N Garrido G Mazola Y Falcón V Torres AM. APL-1, an altered peptide ligand derived from human heat-shock protein 60, selectively induces apoptosis in activated CD4+ CD25+ T cells from peripheral blood of rheumatoid arthritis patients. Int Immunopharmacol (2013) 17:1075–83. doi: 10.1016/j.intimp.2013.10.010 24177275

[83] Konig MF Andrade F. A critical reappraisal of neutrophil extracellular traps and NETosis mimics based on differential requirements for protein citrullination. Front Immunol (2016) 7:461. doi: 10.3389/fimmu.2016.00461 27867381 PMC5095114

[84] Osterloh A Geisinger F Piédavent M Fleischer B Brattig N Breloer M. Heat shock protein 60 (HSP60) stimulates neutrophil effector functions. J Leukoc Biol (2009) 86:423–34. doi: 10.1189/jlb.0109011 19447897

[85] Dapunt U Gaida MM Meyle E Prior B Hänsch GM. Activation of phagocytic cells by staphylococcus epidermidis biofilms: effects of extracellular matrix proteins and the bacterial stress protein GroEL on netosis and MRP-14 release. Pathog Dis (2016) 74(5):ftw035. doi: 10.1093/femspd/ftw035 27109773 PMC5985485

[86] Khandpur R Carmona-Rivera C Vivekanandan-Giri A Gizinski A Yalavarthi S Knight JS. NETs are a source of citrullinated autoantigens and stimulate inflammatory responses in rheumatoid arthritis. Sci Transl Med (2013) 5(178):178ra40. doi: 10.1126/scitranslmed.3005580 PMC3727661 23536012

[87] Maddur MS Miossec P Kaveri SV Bayry J. Th17 cells: biology pathogenesis of autoimmune, inflammatory diseases and therapeutic strategies. Am J Pathol (2012) 181:8–18. doi: 10.1016/j.ajpath.2012.03.044 22640807

[88] Modi S Soejima M Levesque MC. The effect of targeted rheumatoid arthritis therapies on anti-citrullinated protein autoantibody levels and b cell responses. Clin Exp Immunol (2013) 173:8–17. doi: 10.1111/cei.12114 23607804 PMC3694530

[89] Wiersinga WJ Rhodes A Cheng AC Peacock SJ Prescott HC. Pathophysiology, transmission, diagnosis, and treatment of coronavirus disease 2019 (COVID-19): a review. JAMA (2020) 324(8):782–93. doi: 10.1001/jama.2020.12839 32648899

[90] Xu Z Shi L Wang Y Zhang J Huang L Zhang C. Pathological findings of COVID-19 associated with acute respiratory distress syndrome. Lancet Respir Med (2020) 8:420–2. doi: 10.1016/S2213-2600(20)30076-X PMC7164771 32085846

[91] REMAP-CAP Investigators Gordon AC Mouncey PR Al-Beidh F Rowan KM Nichol AD. Interleukin-6 receptor antagonists in critically ill patients with COVID-19. N Engl J Med (2021) 384(16):1491–502. doi: 10.1056/NEJMoa2100433 PMC7953461 33631065

[92] Guimaraes PO Quirk D Furtado RH Maia LN Saraiva JF Antunes MO. Tofacitinib in patients hospitalized with COVID-19 pneumonia. N Engl J Med (2021) 385(5):406–15. doi: 10.1056/NEJMoa2101643 PMC8220898 34133856

[93] Domínguez-Horta MC Venegas-Rodríguez R Guillén-Nieto G Martínez-Donato G Hernández-Cedeño M Bequet-Romero M. CIGB-258, péptido inhibidor de la hiperinflamación en pacientes con COVID-19. Anales la Academia Cienc Cuba (2022) 12(1):e1072.

[94] Centro para el Control Estatal de Medicamentos(CECMED). Jusvinza, emergency use authorization for the treatment of patients with COVID-19 (Autorizo de uso de emergencia a jusvinza, para el tratamiento de pacientes con COVID-19) (2020). Available at: https://www.cecmed.cu/covid-19/aprobaciones/jusvinza-cigb-258-1 (Accessed August 10, 2020).

[95] Centro Nacional de Información de Ciencias Médicas. Protocolo de actuación nacional para la COVID-19 (2020). Biblioteca Médica Nacional. Available at: http://files.sld.cu/bmn/files/2020/08/bibliodir-agosto-2020.pdf (Accessed August 30, 2020).

[96] Mehta P McAuley D Brown M Sanchez E Tattersall R Manson J. COVID-19: consider cytokine storm syndromes and immunosuppression. Lancet (2020) 395(10229):1033–4. doi: 10.1016/S0140-6736(20)30628-0 PMC7270045 32192578

[97] Li G Fan Y Lai Y Han T Li Z Zhou P. Coronavirus infections and immune responses. J Med Virol (2020) 92(4):424–32. doi: 10.1002/jmv.25685 PMC7166547 31981224

[98] Dienz O Rincon M. The effects of IL-6 on CD4 T cell responses. Clin Immunol (2009) 130:27–33. doi: 10.1016/j.clim.2008.08.018 18845487 PMC2660866

[99] Choy E. Understanding the dynamics: pathways involved in the pathogenesis of rheumatoid arthritis. Rheumatology (2012) 51(5):v3–11. doi: 10.1093/rheumatology/kes113 22718924

[100] Zhao Z He S Yu X Lai X Tang S Mariya M EA. Analysis and experimental validation of rheumatoid arthritis innate immunity gene CYFIP2 and pan-cancer. Front Immunol (2022) 13:954848. doi: 10.3389/fimmu.2022.954848 35898498 PMC9311328

[101] Gupta S Bi R Kim C Chiplunkar S Yel L Gollapudi S. Role of NF-κB signaling pathway in increased tumor necrosis factor-α-Induced apoptosis of lymphocytes in aged humans. Cell Death Differ (2005) 12(2):177–83. doi: 10.1038/sj.cdd.4401557 15647756

[102] Gupta S Su H Agrawal S Gollapudi S. Molecular changes associated with increased TNF-α-induced apoptotis in naïve (TN) and central memory (TCM) CD8+ T cells in aged humans. Immun Ageing (2018) 15:2. doi: 10.1186/s12979-017-0109-0 29387134 PMC5775550

[103] Yang Z Wang S Li Q Yuming L Maoti W Hongsheng G. Determining SARS sub-clinical infection: a longitudinal seroepidemiological study in recovered SARS patients and controls after an outbreak in a general hospital. Scand J Infect Dis (2009) 41(6):507–10. doi: 10.1080/00365540902919384 19396666

[104] Huang C Wang Y Li X Ren L Zhao J Hu Y. Clinical features of patients infected with 2019 novel coronavirus in wuhan, China. Lancet (2020) 395:497–506. doi: 10.1016/S0140-6736(20)30183-5 31986264 PMC7159299

[105] Rojas JM Avia M Martín V Sevilla N. IL-10: a multifunctional cytokine in viral infections. J Immunol Res (2017) 2017:6104054. doi: 10.1155/2017/6104054 28316998 PMC5337865

[106] Perlman S Dandekar AA. Immunopathogenesis of coronavirus infections: implications for SARS. Nat Rev Immunol (2005) 5(12):917–27. doi: 10.1038/nri1732 PMC7097326 16322745

[107] Diao B Wang C Tan Y Chen X Liu Y Ning L. Reduction and functional exhaustion of T cells in patients with coronavirus disease 2019 (COVID-19). Front Immunol (2020) 1:827(11). doi: 10.3389/fimmu.2020.00827 PMC7205903 32425950

[108] Chen L Long X Xu Q Tan J Wang G Cao Y. Elevated serum levels of S100A8/A9 and HMGB1 at hospital admission are correlated with inferior clinical outcomes in COVID-19 patients. Cell Mol Immunol (2020) 17:992–4. doi: 10.1038/s41423-020-0492-x PMC7332851 32620787

[109] Wang S Song R Wang Z Jing Z Wang S Ma J. S100A8/A9 in inflammation. Front Immunol (2018) 9:1298. doi: 10.3389/fimmu.2018.01298 29942307 PMC6004386

[110] Zheng M Gao Y Wang G Song G Liu S Sun D. Functional exhaustion of antiviral lymphocytes in COVID-19 patients. Cell Mol Immunol (2020) 17:533–5. doi: 10.1038/s41423-020-0402-2 PMC7091858 32203188

[111] Zheng HY Zhang M Yang CX Zhang N Wang XC Yang XP. Elevated exhaustion levels and reduced functional diversity of T cells in peripheral blood may predict severe progression in COVID-19 patients. Cell Mol Immunol (2020) 17:541–3. doi: 10.1038/s41423-020-0401-3 PMC7091621 32203186

[112] Wang W Su B Pang L Qiao L Feng Y Ouyang Y. High-dimensional immune profiling by mass cytometry revealed immunosuppression and dysfunction of immunity in COVID-19 patients. Cell Mol Immunol (2020) 17:650–2. doi: 10.1038/s41423-020-0447-2 PMC7186533 32346099

[113] Qin C Zhou L Hu Z Zhang S Yang S Tao Y. Dysregulation of immune response in patients with COVID-19 in wuhan, China. Clin Infect Dis (2020) 71(15):762–8. doi: 10.1093/cid/ciaa248 PMC7108125 32161940

[114] Gammazza AM Légaré S Lo Bosco G Fucarino A Angileri F de Macario EC. Human molecular chaperones share with SARS-CoV-2 antigenic epitopes potentially capable of eliciting autoimmunity against endothelial cells: possible role of molecular mimicry in COVID-19. Cell Stress Chaperones (2020) 25:737–41. doi: 10.1007/s12192-020-01148-3 PMC7402394 32754823

[115] Hunter CA Jones SA. IL-6 as a keystone cytokine in health and disease. Nat Immunol (2015) 16:448–57. doi: 10.1038/ni.3153 25898198

[116] Sellegounder D Zafari P Rajabinejad M Taghadosi M Kapahi P. Advanced glycation end products (AGEs) and its receptor, RAGE, modulate age-dependent COVID-19 morbidity and mortality. a review and hypothesis. Int Immunopharmacol (2021) 98:107806. doi: 10.1016/j.intimp.2021.107806 34352471 PMC8141786

[117] Farbstein D Levy AP. HDL dysfunction in diabetes: causes and possible treatments. Expert Rev Cardiovasc Ther (2012) 10:353–61. doi: 10.1586/erc.11.182 PMC3332215 22390807

[118] Park KH Jang W Kim KY Kim JR Cho KH. Fructated apolipoprotein a-I showed severe structural modification and loss of beneficial functions in lipid-free and lipid-bound state with acceleration of atherosclerosis and senescence. Biochem Biophys Res Commun (2010) 392:295–300. doi: 10.1016/j.bbrc.2009.12.179 20059975

[119] Park KH Shin DG Cho KH. Dysfunctional lipoproteins from young smokers exacerbate cellular senescence and atherogenesis with smaller particle size and severe oxidation and glycation. Toxicol Sci (2014) 140:16–25. doi: 10.1093/toxsci/kfu076 24798380

[120] Park KH Cho KH. A zebrafish model for the rapid evaluation of pro-oxidative and inflammatory death by lipopolysaccharide, oxidized low-density lipoproteins, and glycated high-density lipoproteins. Fish Shellfish Immunol (2011) 31:904–10. doi: 10.1016/j.fsi.2011.08.006 21906681

[121] Bucala R Makita Z Vega G Grundy S Koschinsky T Cerami A. Modification of low density lipoprotein by advanced glycation end products contributes to the dyslipidemia of diabetes and renal insufficiency. Proc Natl Acad Sci USA (1994) 91:9441–5. doi: 10.1073/pnas.91.20.9441 PMC44828 7937786

[122] Basta G Schmidt AM De Caterina R. Advanced glycation end products and vascular inflammation: implications for accelerated atherosclerosis in diabetes. Cardiovasc Res (2004) 63:582–92. doi: 10.1016/j.cardiores.2004.05.001 15306213

[123] Devaraj S Dasu MR Rockwood J Winter W Griffen SC Jialal I. Increased toll-like receptor (TLR) 2 and TLR4 expression in monocytes from patients with type 1 diabetes: further evidence of a proinflammatory state. J Clin Endocrinol Metab (2008) 93:578–3. doi: 10.1210/jc.2007-2185 PMC2243229 18029454

